# Eager for an innovative path: solving the puzzle of medical dispute resolution in China combined with bibliometric analysis

**DOI:** 10.3389/frhs.2024.1445536

**Published:** 2024-10-02

**Authors:** Han Zhang, Yan Gu, Bo Liang, Yujie Gao, Fu Zhang, Libing Yun

**Affiliations:** ^1^ Department of Forensic Medicine, West China School of Basic Medical Sciences & Forensic Science, Sichuan University, Chengdu, China; ^2^ Law School, Sichuan University, Chengdu, China; ^3^ Key Laboratory of Forensic Pathology, Ministry of Public Security, Guangzhou, China

**Keywords:** medical dispute, alternative dispute resolutions (ADR), people's mediation committee, medical liability insurance, one-stop service, medical risk prevention

## 1 Introduction

Medical disputes between doctors (hospitals) and patients (and/or their family members) related to diagnosis and treatment (1), are an important subject in health care services and need deepened research for problem-solving strategies (2). Considering the negative impact on both physicians and the healthcare system from medical disputes (3), many countries and regions have increasingly made emphasis on perfect handling of mechanism (4). Although litigation as a traditional method to deal with medical disputes is expected to bring justice to all parties, it often suffers from drawbacks such as lengthy processes, high expenses, complicated procedures and so on (2, 5). To achieve conflict resolution without litigation, alternative dispute resolutions (ADR) have been promoted for their benefits and win-win results (6). Moreover, studies have focused on increasingly medico-legal research on medical malpractice and its ensuing legal problems, then raised the need to seek out-of-court dispute solutions (1–7). Therefore, it is imperative to explore a new approach to handle medical disputes for alleviating the gravity of the existing situation.

According to the international tendency for preventing medical disputes (7), third-party mediation was well-established in China with a long history of mediation tradition (2). Gradually, mediation as a pivotal role for resolving medical disputes is typically carried out by independent organizations, which persuades disputing parties to negotiate voluntarily and reach agreements (6). Nowadays, existing studies showed that the Chinese specialized mechanisms for mediating medical disputes encompass people's mediation served as the predominant force, administrative mediation, and judicial mediation (1). Most recently, an analysis of foreign literature has provided evidence that worldwide healthcare communities increasingly emphasize ADR with benefits in disputes relating to compensation for adverse medical events (8). It is valuable for national health authorities to quantitatively describe and explain the approaches of mediation in China following a number of changes to laws and regulations example for “Medical Disputes Prevention and Treatment Regulations”.

## 2 Bibliometric analysis

In order to explore the situation of mediation within the field of medical dispute research, a bibliometric method can be used to evaluate the results of individuals and organizations to obtain information about the current state of the particular field in this study. The quantitative analyze with CiteSpace software was carried to help researchers find and pursue new research directions swiftly, accurately, and effortlessly. At the same time, visualizing additional information can further uncover and present the inherent connections between information, thereby gaining access to more potential insights. The China National Knowledge Infrastructure (CNKI) dataset was chosen to use for the bibliometric analysis. CNKI is a high-quality Chinese public digital literature resource database covering many different fields. After several attempts, the retrieval strategy in this paper was finally determined as TS = “medical disputes”, with the time span from January 2014 to December 2023, and the article type as “Articles” and “Reviews” 1989 valid articles were finally obtained after filtering out those with lower relevance.

**Figure 1:**
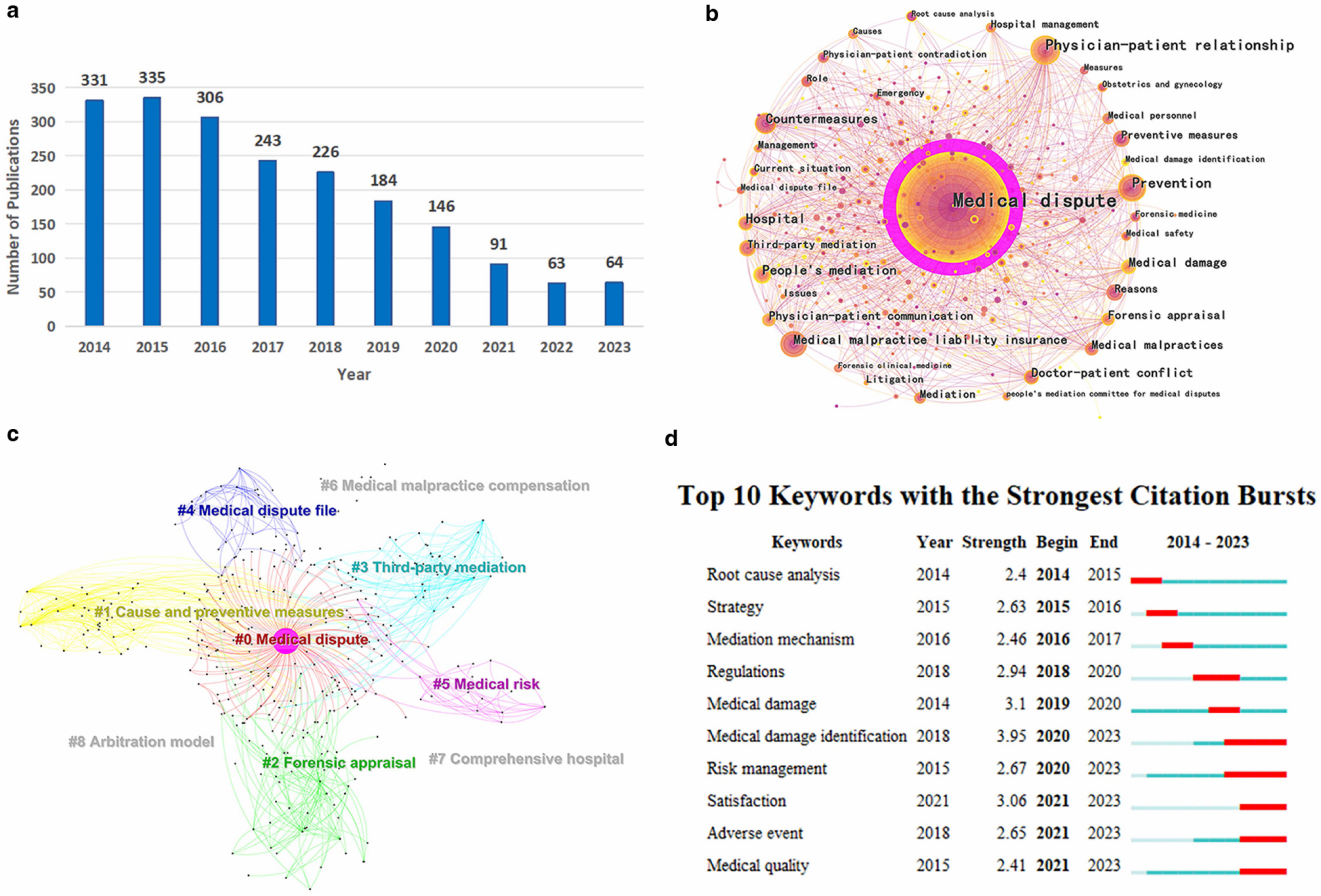
**(a)** The annual number of publications from 2014 to 2023. **(b)** The map of keyword co-occurrence in literatures. **(c)** The network of keyword clusters. **(d)** The map of top 10 keywords with the strongest citation bursts.

It depicts that the number of publications in China peaked in 2015 over the past decade (Figure 1a). This phenomenon can be attributed to the medical healthcare system reform policy implemented in China in 2014. The reform policy emphasized the importance of mechanism construction and aimed at promoting the linkage of medical treatment, medical insurance, and pharmaceuticals through a series of specific measures, improving the quality of medical services, and safeguarding public health. In particular, one of the points proposed to accelerate the development of medical dispute people's mediation and other third-party mediation mechanisms, and to improve the medical dispute resolution and medical risk sharing mechanisms (9). Then, it followed by a slow descent in the number of publications from 2015 to 2022. By utilizing keywords, it can be observed that the themes of these publications are multifaceted, encompassing clinical medicine, forensic medicine, law, insurance and so on (Figures 1b,c). The keyword cluster analysis was performed with the CiteSpace clustering function, and the 8 main clusters are demonstrated (Figure 1c). Among them, cluster #01, #03, #06, #08 belong to law territory, cluster #02 pertains to forensic medicine, while cluster #04, #05, #07, are related to management of hospital. The key word “mediation mechanism” began to burst in 2016 and end in 2017 (Figure 1d), it may be influenced by the promulgation and implementation of the medical healthcare system reform policy implemented in China in 2014. However, as one of the “medical disputes” solutions, the burst growth of “mediation mechanism” is later than the key word “medical disputes” itself.

## 3 Alternative dispute resolution mechanisms in China

In addition to the warm reception of mediation within the academic community, the Chinese government also attaches great importance to the construction of mediation mechanisms. In 2010, China enacted the People's Mediation Law, which upheld the autonomy of people's mediation. The law emphasized the coordination mechanism between people's mediation and other dispute resolution methods, integrating non-litigation mediation paths (10). In 2019, the Ministry of Justice of China proposed to establish a comprehensive multipartite mediation framework according to People's Mediation Law (11). Since then, various regions in China have vigorously promoted the construction of non-litigation dispute resolution mechanisms integrating people's mediation, administration mediation, court mediation, and arbitration (1). It is announced that the multipartite mediation framework has been essentially established in 2022, and research has shown that the standard handling procedures of medical disputes and related compensation rules tailored to local conditions have been formed in some parts of China (2). As the variety of non-litigious dispute resolution mechanisms expands, the public increasingly adopts a rational approach to conflict resolution, eschewing the previous overreliance on contentious methods such as medical disturbances, violent injuries to doctors and the work of complaints and proposals to settle grievances (6).

### 3.1 People's mediation committee for medical disputes with the co-insurance model

In the context of the reform of China's medical and health system initiated in 2014, one of the strategic focuses was the expedited advancement of medical liability insurance (1). It mandated full insurance coverage for all tertiary public hospitals and over 90% for secondary ones, with strong encouragement for non-public hospitals to insure as well (12). Medical liability insurance is a type of insurance that compensates for the economic liability that medical institutions are legally required to bear, according to the contract agreement. Using insurance as a means to establish a third-party pathway and channel for resolving medical liability compensation issues helps patients receive timely financial compensation and fosters a harmonious doctor-patient relationship. Considering an obvious advantage of medical liability insurance in handling medical disputes (3), the co-insurance model has been promoted as better resolutions across various regions in China. Within the framework of coinsurance, multiple insurance companies jointly underwrite the same project by cooperating the premiums and risks based on their insurance coverage (13). What's more, some results highlighted the importance of the connection between the coinsurance and the medical institution facilitated by the insurance broker (3). To be more specific, new form of insurance claims bonded with people's mediation for medical disputes are derived from the third-party mediation mechanism.

Further, this specific way of alternative dispute resolution operated by a certain insurance brokerage company in few Chinese areas is deemed as the design and implementation of the People's Mediation Committee system (14). Beyond the mediation, the People's Mediation Committee establishes a specialized department to build a database to collect and organize mediation cases (14) which is utilized to analyze medical risks and feedback to mediators. When the hospital where the incident occurred performs the high-risk surgery again, the mediator will go to the hospital to conduct a preoperative third-party witnessing session (5). It not only enhances trust and understanding of doctor-patient, but also motivates the hospital and doctors to identify and prevent medical risks (3, 15). Although the above-mentioned model's application received active progress, further investigation on medical risk-sharing mechanism is necessary to provide reliable guidance, such as protection of medical claims and potential legal effectiveness.

### 3.2 One-stop service

When facing conflicts, China always emphasizes the litigation sources governance, which is underpinned by the philosophy that spotlights “prioritizing mediation and reserving adjudication as the final recourse” (11, 16). The one-stop service is a new model of realizing this philosophy. It refers to integrating the medical dispute mediation process into a single platform and the platform provides comprehensive, convenient, and efficient dispute resolution services for both patients and healthcare providers (11). An increasing number of studies have reported the deployment of integrated the one-stop service, which offers multiple dispute resolution with various methods such as mediation, arbitration, litigation and others, alongside associated legal counsel and aid services (17). In certain regions, the People's Courts have integrated tribunals into the one-stop service platforms to facilitate circuit court sessions (18), effectively mitigating the intricacy of the mediation-to-litigation interface procedures to a significant degree. To provide this beneficial services for all parties, the establishment of a centralized platform is based on active supports and cooperation from government (2). Distinguished from other approaches to dispute resolution, the one-stop service are fundamentally dependent on the strength and resources of grassroots organizations (11). Currently, these organizations conducted in various regions suggests distinct advantages of markedly curtailing time and cost, as well as the sustainable advancement of both society and the economy (17). However, some issues referred by publications were raised here, which addressed the persistent challenges including inadequate coordination and shortage of professional force.

## 4 Discussion

Both the Medical Dispute Mediation Committee and the one-stop service were established with the aim of efficiently and conveniently resolving medical disputes. In the light of analytical and evaluative approach achieved, statistical data released by the Ministry of Justice of China showed that over 60% of medical disputes at the end of 2018 are resolved through people's mediation with a high success rate (over 85%) (19). Even more to the point, once the Medical Dispute Resolution Committee has confirmed the liability and compensation amount, the insurance company will compensate the patient more quickly than the hospital, which is a significant advantage of co-insurance model. According to the top ten typical experiences of “one-stop” construction published by the Supreme People's Court, the success rate of mediation in one particular location's the one-stop service exceeded 90% (20). Among the various solutions to medical legal disputes, the one-stop service to both medical providers and patients highlighted these practical procedures for saving time and high efficiency, especially for parties involved in medical disputes with significant disputes.

The database assisting the People's Mediation Committee for Medical Disputes in predicting potential surgical risks in hospitals in advance, contributing to the prevention of medical risks in China. Nevertheless, the preventive components within medical dispute resolution framework in China require further exploration and enhancement to fully realize their potential (5). This opinion article proposes that health department shall be tasked with compiling a comprehensive statistical analysis of medical disputes and constructing an authoritative retrospective case database (21), with the database as part of the data source. Noteworthily, the construction of the database must be conducted with stringent measures to protect patients’ privacy. Sensitive personal data that are extraneous to the medical condition and case specifics, including names, identification numbers, telephone numbers and so on, need to be kept confidential and not disclosed (21). In 2019, the State Council of China highlighted the imperative to significantly enhance the “Internet + Regulation” initiative (22). Similarly, it is worth advocating the government integrating established databases with Hospital Information Systems (HIS) to formulate a medical risk assessment framework by employing big data technologies including machine learning, predictive modeling, neural network learning and so forth (21, 23–25). Collaboratively, an advanced medical risk alert system for medical practices can be established to shift from reactive dispute resolution to proactive prevention, fundamentally reducing the incidence of medical disputes. A research has demonstrated that a province in China has effectively developed and deployed an advanced medical risk alert system, yielding tangible successes to date (26).

While there is growing evidence of bafflement at medical dispute resolution among literature from China and other countries, it is necessary to explore different ways of preventing legal and medical litigation (8). For ensuring transparent communication of surgery details and risks to patients, the preoperative third-party witnessing session mechanism in China is a major supervisory action involving external institutions in conjunction with both hospitals and patients (5). Similar to a culture of openness involved in health system for promoting full transparency in the medical process (27), the independent status of the Healthcare Safety Investigation Branch in UK as an example provides more adequate supervision of medical practices. Studies have shown that higher levels of openness are associated with lower mortality rates (27). Concurrently, some scholars pointed out that an excessive degree of openness may lead to an imbalance between patients’ demands for transparency and the pressure experienced by doctors (28). The development of openness more extensively studied offers important insights for resolving medical disputes in China. A moderate level of openness can safeguard patients’ right to informed consent and ensure a higher degree of supervision over medical actions. Moreover, given China's large population and the burden on hospitals (29), it may not be easy to achieve the balance between the need of patient-physician relationship as the practice of defensive medicine increasing. However, this opinion article has focused that the construction of a supervision system involving massive data disclosure still requires a gradual and cautious approach under China's current government-led system with supervisory powers. Nevertheless, the evolution of the degree and scope of openness remains a valuable reference.

Referencing the deficiencies inherent in the one-stop service, on the one hand, the downward conduction of governance pressures associated with the one-stop service risks creating disconnects at different levels and leading to a loosening of organizational ties (11), thereby highlighting the need for enhanced vertical coordination. On the other hand, the one-stop service have integrated the governance insights of the “Fengqiao Experience” in the new era, a significant litigation source governance approach that mobilizes and relies on the masses and insists on resolving conflicts at the grassroots level (30), but it still necessitates innovative theoretical development and practical application to keep pace with the ongoing law-oriented evolution within the framework of socialism with Chinese characteristics (17, 31). It is vital to clarify the boundaries and interconnections of various diversified dispute resolution methods, and strengthen collaboration with the legal administration and enforcement agencies (17). Moreover, relatively speaking, impressive outcomes from considerable literature focused on proactive measures including information sharing, business coordination and non-convergence mechanisms with the medical authorities (23–26). Particularly, the establishment of a digital platform is essential to facilitate the convenience for the public (32). The public would be able to utilize online services for inquiries, consultations, case registration, progress tracking, fee payment and refunds, and so on, while receiving mediation services offline (33, 34).

With the evolution of sustainable third-party mediation, it is essential to keep the profession of the medical disputes acting or agencies. In fact, the research topics with preventive measure enable us to organize professional assessment panels with skilled personnel because professional mediators from medical practitioners are helpful for all parties to shape a reasonable expectation (15). For example, in Japan, most medical disputes are resolved through the Medical Association, 4 legal and 6 medical experts engage in compensation deliberation and the deliberation decision enables substantial processing of disputes (35). Referring to insightful suggestions following the current study, future research is needed to study the impact of cross-disciplinary coordination and provide greater awareness of the role of general practitioners with more medical and legal knowledge (13, 17, 25, 31).

## 5 Conclusion

Guided by the current challenges in medical disputes, the Chinese government actively sought scientific strategies for resolving conflicts and has explored the construction of a systematic and efficient dispute prevention and resolution system. Successive initiatives such as “multipartite mediation”, “multi-party dispute resolution”, and “litigation source governance” have been implemented to prevent and resolve disputes. The mechanisms have been continuously improved and upgraded, resulting in significant progress in the resolution of medical disputes. Constructing a comprehensive medical dispute resolution system is indeed crucial, however, leveraging collaborative efforts among various participants to bolster the medical risk prevention initiatives within hospitals and doctors to fundamentally eliminate the occurrence of medical malpractice is of paramount importance.
